# Current applications and limitations of European guidelines on blood pressure measurement: implications for clinical practice

**DOI:** 10.1007/s11739-022-02961-7

**Published:** 2022-03-31

**Authors:** Giuliano Tocci, Barbara Citoni, Giulia Nardoianni, Ilaria Figliuzzi, Massimo Volpe

**Affiliations:** Hypertension Unit, Division of Cardiology, Department of Clinical and Molecular Medicine, Faculty of Medicine and Psychology, Sant’Andrea Hospital, University of Rome “Sapienza”, Via di Grottarossa 1035-9, 00189 Rome, Italy

**Keywords:** Home blood pressure, Office blood pressure, Out-of-office blood pressure, Hypertension, Cardiovascular prevention

## Abstract

Hypertension is the most common cardiovascular (CV) risk factor, strongly and independently associated with an increased risk of major CV outcomes, including myocardial infarction, stroke, congestive heart failure, renal disease and death due to CV causes. Effective control of hypertension is of key importance for reducing the risk of hypertension-related CV complications, as well as for reducing the global burden of CV mortality. However, several studies reported relatively poor rates of control of high blood pressure (BP) in a setting of real-life practice. To improve hypertension management and control, national and international scientific societies proposed several educational and therapeutic interventions, among which the systematic implementation of out-of-office BP measurements represents a key element. Indeed, proper assessment of individual BP profile, including home, clinic and 24-h ambulatory BP levels, may improve awareness of the disease, ensure high level of adherence to prescribed medications in treated hypertensive patients, and thus contribute to ameliorate BP control in treated hypertensive outpatients. In line with these purposes, recent European guidelines have released practical recommendations and clear indications on how, when and how properly measuring BP levels in different clinical settings, with different techniques and different methods. This review aimed at discussing current applications and potential limitations of European guidelines on how to measure BP in office and out-of-office conditions, and their potential implications in the daily clinical management of hypertension.

## Introduction

Essential hypertension is a major risk factor for cardiovascular (CV) morbidity and mortality, as well as for hospitalizations due to CV diseases [1]. Despite the beneficial effects demonstrated in treated hypertensive patients who achieve the recommended therapeutic targets in terms of reduced incidence of hypertension-related CV and renal complications [2], proportions of treated hypertensive patients with controlled BP were far from being satisfactory, worldwide [3, 4].

Several reasons have been proposed for explaining the poor rates of BP control achieved under pharmacological therapies. Among these, the relatively low adherence to prescribed antihypertensive therapies [5, 6], the frequent self-adjustments and self-interruptions of prescribed medications [7], the partial or insufficient control of BP levels over the 24 h [8, 9], and the poor awareness of the clinical consequences of high BP [10–14] represent potential pitfalls for preventive strategies aimed at reducing the burden of hypertension-related morbidity and mortality.

To overcome these limitations, recent international guidelines have strongly reaffirmed the importance of proper BP measurements, to improve awareness of the disease, ameliorate therapeutic adherence, and ensure high rate of BP control in treated hypertensive outpatients [15–18]. The achievement of these goals has been facilitated by the widespread diffusion of automated and semi-automated devices for home (HBP) and 24-h ambulatory BP (ABP) measurements over the last few years. Indeed, the progressive implementation of validated, low-cost and comfortable devices for measuring out-of-office BP ameliorated physicians’ ability in tailoring antihypertensive therapies to individual characteristics and CV risk profile, and facilitated their attitude to properly and early identify some specific hypertension patterns at higher risk of CV events compared to normotension, including white-coat hypertension (WCHT) [19, 20] and masked hypertension (MHT) [21, 22].

Given the importance of proper BP assessment in the daily clinical practice, this review is aimed at discussing current applications and potential limitations of European guidelines on how to measure BP in office and out-of-office conditions, and their potential implications in the daily clinical management of hypertension.

## Office and out-of-office BP measurements

According to recommendations from current guidelines [15–18], BP levels can be measured in different clinical settings, in different positions, with different devices and according to different protocols.

Independently of the setting, the devices or the protocols adopted for measuring BP levels, proper cuff sizes must be applied, depending on arm circumference of individual patients (small 6–11 cm, small/medium 10–19 cm, medium 18–26 cm, large 22–32 cm and extra-large 33–47 cm). Adoption of improper cuff size still represents one of the most frequent causes of misdiagnosed hypertension in a setting of clinical practice. In this view, recent availability of conic cuffs for obese patients has represented a further improvement in diagnostic facilities for proper hypertension diagnosis and control [23, 24].

### Office BP measurement

**Fig. 1:**
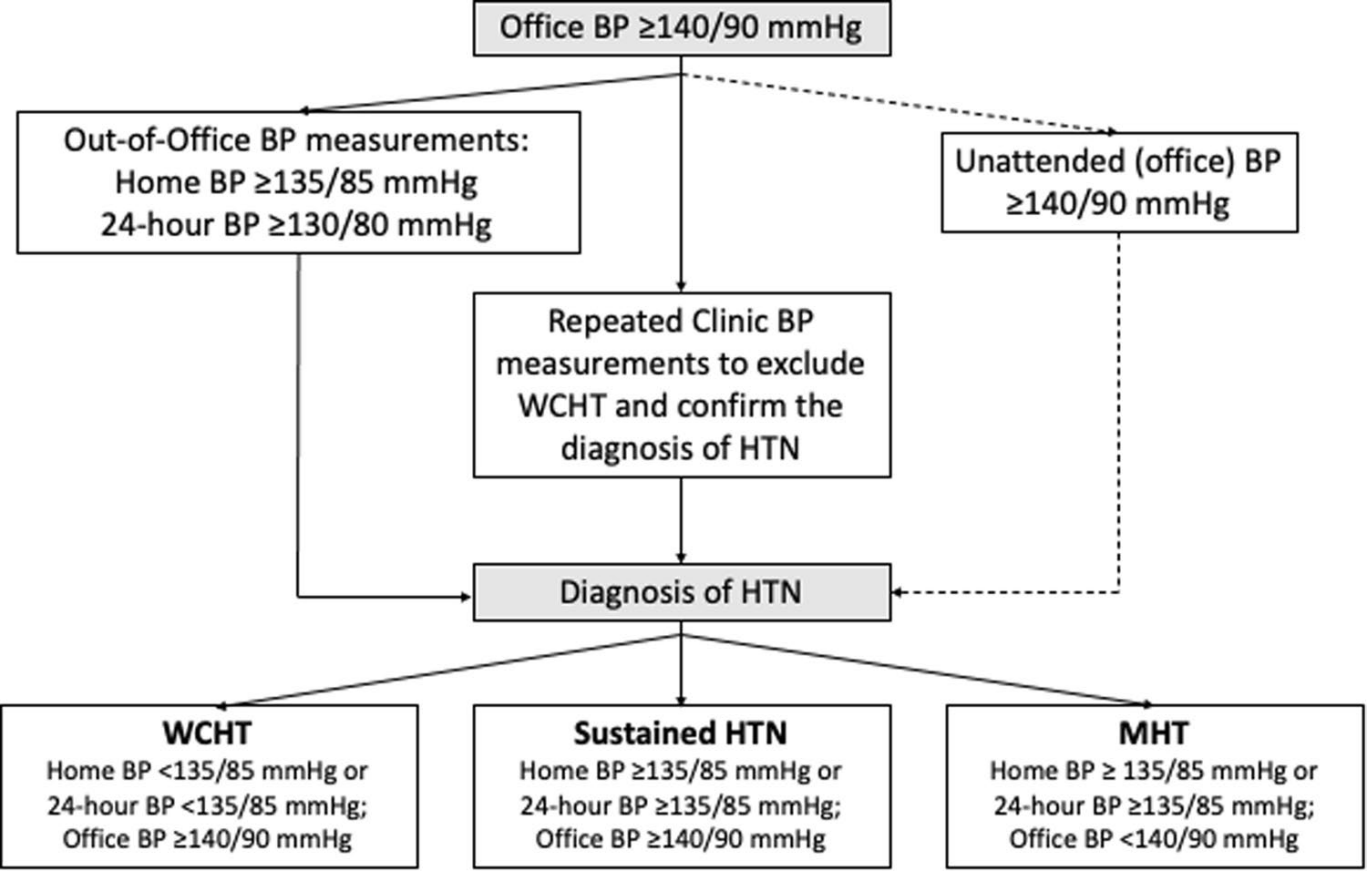
Schematic representation of the current diagnostic approach to hypertension, as recommended by current guidelines. Office and out-of-office BP measurements are currently recommended by European guidelines to confirm the diagnosis of hypertension, as well as to properly identify different hypertension phenotypes. Unattended (office) BP measurements have been recently proposed as a valid option in the diagnostic work-up of hypertension. *BP* blood pressure, *HTN* hypertension, *WCHT* white-coat hypertension, *MKHT* masked hypertension

Office (clinic) BP measurement still represents the gold standard for confirming the diagnosis of hypertension and evaluating the clinical effectiveness of a given treatment in hypertension [15–18], as schematically illustrated in Fig. 1. Classification of hypertension in different grades is based on office BP levels and antihypertensive treatment can be tailored in individual patients according to these BP measurements [15–18].

Office BP levels should be measured in a quiet room, after 3–5 min of rest, avoiding any interference by external factors or stressors. During office BP measurement, mobile phones and other electronic devices should be turned off or set on “flight mode” and patient should stay in a conformable position with the upper arm supported at the heart level. If the upper arm is below the level of the right atrium, the BP readings may be too high; if the upper arm is above heart level, the BP readings may be too low. If the arm is unsupported and held up by the patient, BP levels may result higher than true values. Afterward, sequential BP measurements should start, and the average of three consecutive BP measurements and heart rate has to be considered as clinic (office) systolic/diastolic BP levels [16].

Office BP measurement can be performed with the patient in the sitting position (seated BP) or in the supine position (supine BP). In frail or elderly individuals or in those with clinical suspicion of orthostatic hypotension, BP should be measured also in the upright position (standing BP). A BP drop of 20 mmHg in systolic BP or a drop of 10 mmHg in diastolic BP within 2–5 min of standing, or if standing causes signs and symptoms should be considered as a diagnostic marker of orthostatic hypotension.

During screening assessment, office BP levels should be measured at both arms. Then, in the absence of significant BP difference between arms, the same arm should be used for consecutive BP measurements during the follow-up visits.

Periodic office BP assessment can be made for confirming the diagnosis of hypertension, according to the recommendations of current guidelines [15]: at least every 5 years in those individuals with optimal BP (< 120/80 mmHg), at least every 3 years in those with normal BP (120–129/80–85 mmHg), and at least every year in those with high-normal BP (130–139/85/89 mmHg). In this latter case or in the presence of high/very high CV risk or unexplained hypertension-mediated organ damage (HMOD), out-of-office BP measurements can be used to confirm or exclude the diagnosis of hypertension [15].

### Out-of-office BP measurements

Out-of-office BP assessment includes HBP and 24-h ABP monitoring. There are specific clinical indications for both these BP measurements in a setting of clinical practice, as reported in Table 1 [16]. These indications, as well as potential clinical limitations or contra-indications, should be always considered before prescribing or modifying any antihypertensive medications.

**Table 1:** Indications, contra-indications, advantages and disadvantages of different types of office and out-of-office BP measurements

	Office attended BP	Office unattended BP	Ambulatory BP	Home BP
Indications	To confirm the diagnosis of hypertension	To obtain BP levels less affected by WCH phenomenon	To detect WCHT and MHT	Long-term follow-up of treated hypertension
	To evaluate the clinical effectiveness of a given treatment in hypertension		To identify nocturnal hypertension and non-dippers	
	To classify hypertension in different grades		To assess BP changes due to autonomic failure	
			To ensure 24-h BP control	
Contraindications	Will not detect MHT	Will not detect MHT		
Advantages	Readily available in most healthcare settings	Reduces, but does not eliminate the WCH phenomenon	Objective results over 24 h	Widely available at relatively low cost
	Strong data linking OBP with CVD	Typically give lower values than usual OBP measurements, which appear to be similar to day-time ABPM		
	Used in most observational and interventional outcome trials in hypertension			
			Detects WCHT and MHT	Acceptable to patients for long-term use
			Confirms uncontrolled and RHT	Detects WCHT and MHT
			Assesses BP during usual daily activities	Confirms uncontrolled and RHT
			Detects nocturnal hypertension and non-dippers	Detects excessive BP lowering from drug treatment. Improves adherence with treatment and thereby hypertension control rates
			Detects excessive BP lowering by drug treatment	Can reduce healthcare costs
Disadvantages	Inadequate reproducibility, with single-visit OBP having low diagnostic precision in an individual	The MHT phenomenon is present as with usual OBP measurements	Rather expensive and time-consuming for healthcare provider	Inaccurate devices and inappropriate cuff size often used
	Subject to WCH (reduced but still present with standardized measurements taken in repeated visits)	Unattended OBP measurement may not be feasible in several settings in clinical practice	May cause discomfort particularly during sleep	Monitoring may be too frequent, in the presence of symptoms, and under inappropriate position
	Will not detect MHT	The threshold for diagnosing hypertension using unattended OBP is yet not clearly defined and with insufficient outcome data	Suboptimal reproducibility for diagnosis within 24 h	May induce anxiety to some patients
		Data on the relationship between unattended office BP and CV events are much less solid as compared to those obtained with the standard approach based on attended office BP	Asleep BP often not calculated using the individuals’ sleeping times	Risk of unsupervised treatment changes by patients

Derived from Reference num. [16]

#### Home BP measurements

HBP measurements can be performed with automatic or semi-automatic devices, the majority of which are electronic (oscillometric) monitors; traditional (auscultatory) sphygmomanometers are not recommended for HBP monitoring, due to the required expertise [16]. Whatever the case, it is fundamental that patients adopt validated devices for measuring HBP [15–18]. For the clinical validation of electronic BP monitors, several protocols developed by scientific organizations have been proposed over the last years. All guidelines recommend that only devices successfully validated by a given protocol should be used [15–18]. Unfortunately, most of the devices available on the market have not been subjected to independent evaluation using an established protocol.

Before clinical consultation, HBP levels should be measured twice in the morning and twice in the evening for 7 consecutive days [16]. During the follow-up period, HBP can be measured once or twice per week with at least two repeated measurements in the morning or in the evening; the minimum request is having at least two repeated measurements per month. In the morning HBP should be measured before breakfast and before assumption of any antihypertensive therapy, if applicable; in the evening, it should be preferably measured before dinner. Whatever the case, it should be always measured in normal conditions, avoiding physical or emotional stressors that may affect or disturb the BP assessment. All BP (and heart rate) values should be reported in a specifically designed BP diary, as that proposed by the guidelines [16].

In the recent years, the availability of electronic BP monitors able to share data with mobile phones and health Apps (mHealth) has markedly boosted. This will lead to a rapid improvement in the use of telemedicine for monitoring home BP levels of treated and untreated hypertensive patients [25], particularly in case of physical inability or home isolation, as widely observed during the COVID-19 pandemic [26]. The clinical effectiveness of this approach has to be confirmed, though it probably represents one of the most attracting innovations for ameliorating hypertension awareness and control in the real world [27, 28].

In this latter regard, it should be noted that uncontrolled use of telemedicine and mHealth solutions out of medical surveillance may encourage patients' self-medications or self-adjustments (or even interruptions) of prescribed antihypertensive medications, with potentially deleterious consequences on effective BP control and possibly increased risk of hypertension-related CV events. Thus, general practitioners and reference physicians should be well aware of this aspect, when adopting telemedicine, and be available for remote consultations, when necessary. A recent international consensus document has been highlighted all these aspects and recommended a standardized approach for the use of telemedicine in the clinical management of hypertension [27].

#### Ambulatory BP measurements

24-h ABP monitoring is performed with oscillometric devices. The device should be set in the outpatient clinic after completion of the office BP measurements. Each patient should be instructed not to alter her/his usual schedule during the monitoring period, asked to avoid unusual physical activities, and to maintain the arm still during BP measurements.

Automatic BP readings are obtained every 15 min during the day-time period and every 30 min during the night-time period over the 24 h [16]. It should be noted, however, that different time intervals between the readings have been adopted in different centers and in clinical studies [29–31], and these are not rejected by current [16] and previous [32] guidelines. In addition, depending on the adopted devices and BP monitors, some centers have the possibility to modify predefined time intervals for day-time (from 6:00 AM to 22:00 PM) and night-time (from 22:00 PM to 6:00 AM) periods, on the basis of activities reported in patients’ own diaries. In particular, night-time period may be adapted, depending on how long the patient stays in bed (total night-time period in supine position), how long she/he actually sleeps (real night-time period), and when she/he gets up from bed (asleep day-time period).

Average values for the 24-h, day-time and night-time systolic and diastolic ABP levels (and heart rate) can be extracted. In addition, standard deviations from average values, as well as number of BP measurements above the normal BP thresholds were reported for each time period (24-h, day-time and night-time) in each participant.

### Hypertension phenotypes

Essential hypertension can be further characterized into four BP phenotypes by combining information derived from the assessment of office and out-of-office (either home or 24-h ambulatory) BP measurements [15–18]. Specifically, normotension was defined by the presence of both office and 24-h ABP levels below the normal thresholds of < 140/90 mmHg and < 130/80 mmHg, respectively. WCHT (or isolated clinic hypertension) is defined by the presence of abnormal office (≥ 140/90 mmHg) and normal 24-h (< 130/80 mmHg) BP levels, whereas MHT (or reverse white-coat hypertension) is defined in the presence of normal office (< 140/90 mmHg) and above normal 24-h (≥ 130/80 mmHg) BP levels. Finally, sustained hypertension is defined when both office and 24-h BP levels are above the normal thresholds of ≥ 140/90 mmHg and ≥ 130/80 mmHg, respectively. A schematic representation of this classification is illustrated in Table 2 [16].

**Table 2:** Curt-off values from the diagnosis of different hypertension phenotypes, including normotension (NT), white-coat hypertension (WCHT), masked hypertension (MHT), and sustained hypertension (SHT)

	Office BP < 140/90 mmHg	Office BP > 140/90 mmHg
24-h BP < 130/80 mmHg	normotension	WCHT
24-h BP > 130/80 mmHg	MHT	SHT

Derived from Reference num. [16]

This classification was originally proposed for screening purposes in untreated, naïve individuals with or without BP elevations. Later on, however, several clinical studies have extended these definitions also to treated hypertensive outpatients, suggesting the following hypertension categories, respectively: treated controlled hypertension, clinic uncontrolled hypertension, masked uncontrolled hypertension, and sustained uncontrolled hypertension [33]. As expected, the risk of having hypertension-related CV complications was higher in those individuals with prolonged BP elevations (masked or sustained hypertension) than in those with time-limited BP raise, both in the presence or in the absence of antihypertensive medications.

This latter point should be always considered, when approaching patients with documented evidence of WCHT or MHT, since both these conditions have been associated with an increased risk of major CV outcomes, especially in the presence of metabolic risk factors (e.g., hypercholesterolemia, obesity, metabolic syndrome, and diabetes mellitus) or markers of hypertension-related organ damage. Thus, they should not be considered fully benign, yet they indeed deserve closer clinical observation and proper control of additional risk factors and comorbidities.

### White-coat hypertension

White-coat hypertension is a clinical condition characterized by an elevation in office BP but normal HBP or ABP values [15, 16]. Sustained BP elevation during and soon after clinical consultation properly defines the presence of WCHT (isolated clinic hypertension), which differs from the abnormal BP rise (often associated with sinus tachycardia and other symptoms) observed in the presence of the “white-coat effect”. This latter is frequently induced by abnormal sympathetic nervous activation and is a time-limiting phenomenon, which might be attenuated by repeated BP measurements during the visit. Although WCHT and white-coat phenomenon can be observed at all hypertension stages, they are more frequent in those with stage 1 hypertension and in those with apparently resistant hypertension.

HMOD is less prevalent in WCHT than in MHT or in sustained hypertension, and recent clinical studies demonstrated that the risk of CV events associated with WCHT is also lower than that in sustained hypertension, though the risk of hospitalizations due to hypertension is higher than those observed in MHT [34]. On the other hand, compared to normotension, patients WCHT have increased BP variability [35], higher prevalence of metabolic risk factors, cardiac and vascular HMOD [36], and a greater long-term risk of new-onset diabetes [37, 38].

The diagnosis of WCHT should be confirmed by repeated office and out-of-office BP measurements, and should include an extensive assessment of risk factors and HMOD [15, 16]. Both HBP and 24-h and ABP measurements are recommended to confirm WCHT [15, 16].

### Masked hypertension

Masked hypertension (MHT) is a clinical condition characterized by normal BP levels when measured during clinical consultation and above normal BP levels at HBP or 24-h ABP monitoring [15, 16]. The presence of MHT has been initially revealed throughout the systematic evaluation of full BP profile, including office, HBP and 24-h ABP levels, in large epidemiological surveys and observational clinical studies in hypertension performed during the last few years [30, 36, 39, 40]. These studies have consistently shown that, despite normal office BP values, MHT is associated with an increased risk of developing HMOD [36, 41, 42], as well as to an increased risk of CV events [37, 43]. However, several items related to this condition are still debated, among which definition, diagnostic criteria, prevalence, and clinical implications in the setting of clinical practice [44–47].

These controversial aspects can be explained by the following factors: (1) it is still not fully accepted if the definition of MHT should be limited to untreated individuals or can be applied also to treated hypertensive patients [48–50]; (2) there is no agreement on which BP thresholds should be considered as out-of-office BP levels (i.e., HBP, 24-h, day-time, night-time, or all periods) [51]; (3) there are opposing data on its estimated prevalence, due to the potential selection bias related to the previous point; (4) there is still limited evidence in favor of the potential benefits obtained by treating normal/high-normal clinic BP levels in MHT patients in terms of reduced incidence of CV events.

### Attended and unattended BP measurements

In the recent years, it has been suggested the adoption of a novel approach for measuring office BP levels without the presence of physicians or nurses, by leaving the patient in seated position in a quiet room for a given time interval (at least 5–10 min) and then automatically initiating three-to-five office BP measurements with 1-min interval between consecutive measurements. In some studies, two additional BP measurements in standing position have been performed.

This approach, called “unattended BP” or “automated office BP”, was first adopted in the Systolic Blood Pressure Intervention Trial (SPRINT) trial [52], which compared the benefit of treatment of systolic BP to a target of less than 120 mmHg with treatment to a target of less than 140 mmHg. The trial was early interrupted after a median follow-up of about 3 years, due to a significantly lower rate of the primary composite outcome (myocardial infarction, acute coronary syndromes, stroke, heart failure, or death from CV causes) in the intensive-treatment group than in the standard-treatment group. The results, however, raised some concerns and criticisms just because the adoption of “unattended” office BP instead of the conventional “attended” office BP measurements [53–55].

Indeed, the main advantages of unattended BP seem to be: (1) the mitigation of white-coat phenomenon; (2) application of a rigorous and reproducible operator-independent protocol for measuring BP in office, though in a comfortable condition similar to that obtainable at home; (3) availability of BP levels closer to individual patients’ BP profile (i.e., similar to home or day-time BP). In view of these characteristics, it has been suggested that measurement of unattended BP values may provide advantages over conventional BP measurement [56–58]. In line with these considerations, some international guidelines now suggest unattended BP as the preferred approach for measuring BP [17, 59, 60].

Data on the relationship between unattended office BP and CV events are much less solid as compared to those obtained with the standard approach based on attended office BP, and this was probably due to the limited number of available clinical studies. Preliminary data, however, suggested that both attended and unattended BP are related with cardiac [61] and vascular [62] HMOD.

Although with some caution due to partial heterogeneity among studies, unattended BP can be considered a valid option for measuring BP in office by limiting or eliminating the white-coat phenomenon [56, 63]. On the other hand, it may be more time-consuming and require available dedicated rooms.

## Practical considerations for choosing among different blood pressure measurements

The best choice among the different types of BP measurements, techniques and protocols in a setting of daily clinical practice largely depends on what doctors want to know and which is the clinical hypothesis. Indeed, each type of BP measurements has specific advantages, disadvantages, costs, limitations, and contra-indications, as schematically represented in Table 3.

**Table 3:** Strengths and limitations of different types of office and out-of-office BP measurements

	Office attended BP	Office unattended BP	Home BP	Ambulatory BP
Availability	+ + +	+	+ + +	+ +
Reproducibility	+ +	+ +	+	+ +
Multiple readings	+	+	+ + + +	+ + +
WCHT phenomenon	+ + +	+	+	+ +
Comfort	+ +	+ + +	+ + +	+
Cost	+	+ +	+	+ +
Correlation with HMOD	+ + + +	+ + + +	+ +	+ +
Correlation with MACE	+ + + +	+	+ +	+ + +
Evidence from RCTs	+ + + +	+	+	+ +

It should be always considered that clinic BP assessment still represents the gold standard for both diagnostic and therapeutic purposes in asymptomatic individuals with clinical suspicion of high BP, as well as in treated hypertensive outpatients. Furthermore, risk stratification in hypertension is still largely linked to office attended BP measurement. The opportunity to integrate data derived from clinic BP measurement with out-of-office BP measurements, such as home BP or 24-h ambulatory BP, should be strongly encouraged.

Home BP monitoring has been adopted in several clinical studies, which not only demonstrated a strong and positive correlation with HMOD and CV outcomes [64–67], but also reported a high level of patients’ awareness and therapeutic adherence to prescribed medications [28, 64, 68]. It should be noted, however, that there is no evidence from randomized controlled clinical trials for initiating or titrating antihypertensive medications based on home BP levels. In addition, the clinical effectiveness of this technique might be impaired by patients’ interferences (for example, patients can alter their own BP diaries by reporting false or discontinuous BP measurements). In this view, the recent availability of telemedicine may provide a valid tool to overcome some intrinsic limitation of this approach, by self-reporting and sharing BP data from patients’ devices to treating physicians or hypertension centers without direct interventions of healthcare personals or of the patients or care-givers.

To limit patients’ interferences and ensuring higher levels of accuracy, the recent adoption of clinic unattended BP measurements seems to provide a very useful and attractive alternative. This approach has, in fact, the potential advantages of combining the rigorous protocol of clinic BP assessment and the advantages of a comfortable and quiet room, in the absence of physicians or nurses [56, 63].

Out-of-office BP measurement can be integrated using 24-h ambulatory BP monitoring. Also in this case, solid clinical evidence is available to support its use for both confirming the diagnosis of essential hypertension and other hypertension phenotypes (WCHT and MHT), and for tailoring antihypertensive therapies for ensuring effective and sustained BP control over the entire 24-h period. In view of the standardized methodology applied for measuring BP levels, 24-h ambulatory BP monitoring might be preferred over before initiation of therapy, as well as in patients stabilized by therapy. Patients, however, might experience some discomfort from repeated BP measurements, mostly during the night-time period, with obvious consequences on the proper interpretation and reproducibility of this technique [69]. A potential solution for this problem would be the use of modern devices for measuring 24-h ABP levels, which adopted cuff-less methods or other wearable leads applied on smartphones and other electronic devices. The applicability and accuracy of these devices in a setting of clinical practice have to be proven.

## Conclusions

Proper assessment of office BP levels represents the first, fundamental step to confirm the diagnosis of hypertension, which still represents the major contributor for CV morbidity and mortality worldwide. Recent guidelines have, for the first time, recommended the implementation of out-of-office BP measurements, both HBP and 24-h ABP, as a valid tool during the diagnostic work-up of hypertension, as well as for properly identifying specific hypertension phenotypes, such as WCHT and MHT. On the other hand, both office and out-of-office BP measurements can be used for tailoring antihypertensive strategies and evaluating the clinical effectiveness of antihypertensive medications during the entire 24-h period.

This comprehensive approach, suggested by guidelines and based on full (office, home and 24-h) BP profile, has several clinical advantages, among which the improvement of the overall rates of BP control, the increase in awareness and adherence to prescribed antihypertensive medications, and, thus, the reduction of hospitalizations due to hypertension crisis or other acute CV events. In line with these purposes, systematic assessment of HMOD and individual CV risk profile should be always considered, beyond definition of hypertension phenotype, to ameliorate diagnostic accuracy and patients’ prognosis.

In the next future, the availability of mHealth technologies and other electronic facilities for telemedicine will probably induce a further and rapid expansion of hypertension assessment, by promoting earlier diagnosis of asymptomatic hypertension and hopefully a better BP control than previously reported in treated hypertensive outpatients. The future tasks are represented by a more extended and reproducible use of these innovations for reducing the incidence of hypertension-related CV diseases and complications.
